# Are the Early Postoperative Outcomes of Coronary Artery Bypass Grafting Surgery in Elderly Women Worse Compared to Men's?

**DOI:** 10.21470/1678-9741-2016-0071

**Published:** 2017

**Authors:** Ahmet Yüksel, Irem Iris Kan, Atıf Yolgösteren, Yusuf Velioğlu, Mustafa Çagdaş Çayır, Orçun Gürbüz, Gencehan Kumtepe, Serkan Akarsu, Murat Biçer, Mustafa Tok, Işık Şenkaya

**Affiliations:** 1 Department of Cardiovascular Surgery of Bursa State Hospital, Bursa, Turkey.; 2 Department of Cardiovascular Surgery of Uludag University Faculty of Medicine, Bursa, Turkey.; 3 Department of Cardiovascular Surgery of Abant Izzet Baysal University Faculty of Medicine, Bolu, Turkey.; 4 Department of Cardiovascular Surgery of Balıkesir University Faculty of Medicine, Balıkesir, Turkey.

**Keywords:** Coronary Artery Bypass, Women, Aged

## Abstract

**Objective:**

To investigate the impact of gender difference in early postoperative outcomes in elderly patients (aged 70 or older) undergoing coronary artery bypass grafting surgery.

**Methods:**

Between October 2009 and December 2013, a total of 223 elderly patients (aged 70 or older) undergoing isolated primary coronary artery bypass grafting surgery were included in this retrospective observational cohort study. Patients were divided into two groups according to their gender. The patients' medical records were collected, their baseline preoperative characteristics, operative data, and postoperative outcomes were retrospectively reviewed, and the effect of gender difference in the early postoperative outcomes was analyzed.

**Results:**

Group 1 (female patients) and Group 2 (male patients) consisted of 71 and 152 patients, respectively. Mean age of patients was 74.4±3.6 years (range: 70-84 years). The level of EuroSCORE I, the incidence of hypertension and hyperlipidemia were significantly higher in Group 1, while the rate of smoking was significantly higher in Group 2. Mean postoperative intubation time, length of intensive care unit and hospital stay were longer in female patients than in male patients, but these differences were not statistically significant. No statistically significant difference between two groups in terms of the transfusion of blood products was observed. The rates of in-hospital mortality and major postoperative complications were statistically similar between the two groups.

**Conclusion:**

In conclusion, the female gender was not associated with worse early postoperative outcomes in elderly patients undergoing coronary artery bypass grafting surgery.

**Table:** 

Abbreviations, acronyms & symbols	
CABG	= Coronary artery bypass grafting
CPB	= Cardiopulmonary bypass
CI	= Confidence interval
DM	= Diabetes mellitus
EuroSCORE I	= European System for Cardiac Operative Risk Evaluation I
HL	= Hyperlipidemia
HT	= Hypertension
ICU	= Intensive care unit
LITA	= Left internal thoracic artery
OPCAB	= Off-pump coronary artery bypass grafting
SPSS	= Statistical Package for Social Sciences

## INTRODUCTION

Female gender is considered to be an independent predictor of morbidity and mortality following coronary artery bypass grafting (CABG)^[1-3]^. Previous studies showed that the risk of operative mortality after isolated CABG was approximately 1.5-2 fold higher in women than in men^[1,4-6]^. Many investigators have been interested in and pondered over the influence and outcomes of gender difference with regard to CABG for a long time. The most considerable and accepted opinion on this topic is that female patients undergoing CABG surgery are greater-risk patients than males. Different causes have been revealed to clarify why women have greater-risk following CABG surgery than men, including later onset of coronary artery disease and older age, smaller body size and coronary artery diameters, under-utilization of arterial grafts, and more existence of comorbid conditions such as diabetes mellitus (DM) and hypertension (HT)^[6,7]^. In fact, these factors may be associated with poorer postoperative outcomes and confound any comparison between two genders.

There is also inconsistency between gender difference and postoperative outcomes following CABG in the previous studies when examined and compared with each other. In many studies female gender was found associated with worse postoperative outcomes after CABG surgery^[8-11]^, while in some other studies which especially had the propensity-matching for the adjustment of risk factors, demonstrated that the outcomes in females were similar to their matched male counterpart following CABG^[12-15]^. Thus, the impact of gender difference on postoperative outcomes after CABG is a debated topic of ongoing relevance. Moreover, the information about the impact of gender on postoperative outcomes following CABG in elderly patient is limited. Therefore, this study aimed to investigate the impact of gender difference in early postoperative outcomes in elderly patients (aged 70 or older) undergoing CABG.

## METHODS

### Study Design

The study population consisted of 1087 patients who underwent elective isolated first-time CABG in our institution between October 2009 and December 2013. Among them, 223 (20.5%) patients were aged 70 or older, and included in this study, following the approval of institutional ethics committee. Patients were divided into two groups according to their gender. Group 1 (female patients) consisted of 71 patients (mean age: 74.3±3.4 years) and Group 2 (male patients) consisted of 152 patients (mean age: 74.4±3.7 years). The medical data were collected, and baseline characteristics, intraoperative and postoperative records were retrospectively analyzed. Patients undergoing emergency surgery, redo surgery, concomitant valvular surgery, left ventricular surgery, or other cardiovascular procedures were excluded.

### Surgical Approach

The surgical approach to be used was decided one day before the operation. The final decision was given after evaluation of coronary anatomy and the palpation of the aorta during operation. All cases were operated under general anesthesia and via median sternotomy. Left internal thoracic artery (LITA) and great saphenous vein were mainly used as bypass grafts in majority of patients. Pedicled LITA harvesting technique was routinely preferred in all of patients with LITA graft. In Group 1, 32 patients underwent off-pump coronary artery bypass grafting (OPCAB), while 39 patients had conventional CABG with cardiopulmonary bypass (CPB). In Group 2, 57 patients had OPCAB, while 95 underwent conventional CABG.

### Postoperative Follow-Up

Postoperative intensive care unit (ICU) follow-up was standardized for all patients. All patients received intravenous nitroglycerin for the first 24 hours unless hypotension (systolic blood pressure < 90 mmHg). Selection of inotropic agents was dictated by the hemodynamic data. Patients were transferred from ICU to clinical service when they were hemodynamically stable. Some routine medications included daily oral acetylsalicylic acid, and beta blockers unless hypotension and bradycardia (systolic blood pressure < 90 mmHg, heart rate < 60/min). Angiotensin converting enzyme inhibitors, diuretics, digoxin, and warfarin were gradually introduced when clinically indicated.

### Statistical Analysis

Continuous variables were expressed as mean ± standard deviation. Categorical variables were expressed as frequency and percentages. While comparing the two groups, Student's t-test was performed for the continuous variables, and Chi-squared test was performed for the categorical variables. Two tailed *P* values < 0.05 were considered as significant, and confidence interval (CI) was 95%. All statistical analyses were performed using the Statistical Package for Social Sciences (SPSS) program (version 18.0, SPSS, Chicago, IL, USA).

## RESULTS

Demographic and baseline clinical characteristics of patients are summarized in Table 1. Mean age of the study population was 74.4±3.6 years (range: 70-84 years). The mean level of the European System for Cardiac Operative Risk Evaluation I (EuroSCORE I), history of HT and hyperlipidemia (HL) were significantly higher in female patients when compared to males (*P*<0.05). The rate of smoking was significantly lower in women. The groups were statistically similar in terms of the other demographic and comorbid features.

**Table 1:** Demographic and baseline clinical characteristics of the groups.

	Group 1 (Female)	Group 2 (Male)	*P* value
Overall (n, %)	71 (31.8%)	152 (68.2%)	
Age, years (mean±SD)	74.3±3.4	74.4±3.7	0.82
LMCA disease (n, %)	12 (16.9%)	28 (18.4%)	0.85
Ejection fraction <50% (n, %)	22 (31.0%)	36 (23.7%)	0.27
EuroSCORE I level (mean±SD)	6.3±3.0	5.1±2.4	0.027
CCS class for angina (mean±SD)	1.68±0.93	1.62±0.87	0.42
BMI, kg/m^2^ (mean±SD)	27.6±4.4	27.4±4.1	0.74
Hypertension (n, %)	60 (84.5%)	107 (70.4%)	0.031
Diabetes mellitus (n,%)	27 (38.0%)	40 (26.3%)	0.06
Hyperlipidemia (n,%)	40 (56.3%)	57 (37.5%)	0.009
Family history (n,%)	8 (11.3%)	11 (7.2%)	0.17
Smoking (n,%)	8 (11.3%)	54 (35.5%)	0.000
PAD (n,%)	6 (8.5%)	9 (5.9%)	0.37
COPD (n,%)	4 (5.6%)	11 (7.2%)	0.44
Renal dysfunction (n,%)	5 (7.0%)	13 (8.6%)	0.59
Chronic liver disease (n,%)	1 (1.4%)	1 (0.7%)	0.15
History of CVE (n,%)	6 (8.5%)	10 (6.6%)	0.67
History of previous PCI (n,%)	6 (8.5%)	16 (10.5%)	0.81

BMI=body mass index; CCS=Canadian Cardiovascular Society; COPD=chronic obstructive pulmonary disease; CVE=cerebrovascular event; EuroSCORE=European System for Cardiac Operative Risk Evaluation; LMCA=left main coronary artery; PAD=peripheral arterial disease; PCI=percutaneous coronary intervention; SD=standard deviation

Intraoperative data and postoperative clinical outcomes of patients are presented in Tables 2 and 3. There were no statistically significant differences when compared both groups according to the intraoperative data. Mean postoperative intubation time, length of ICU and hospital stay were longer in female patients than in male patients, but these differences were not statistically significant. No statistically significant differences between the two groups in terms of the transfusion of blood products were found. The occurrence of major postoperative complications, including low cardiac output syndrome, postoperative myocardial infarction, cerebrovascular events, respiratory failure, reexploration for bleeding, mediastinitis, new onset of acute renal failure were statistically similar between two groups. In-hospital mortality rates of both groups were also statistically similar.

**Table 2:** Intraoperative data of the groups.

	Group 1 (Female)	Group 2 (Male)	*P* value
Off-pump CABG (n, %)	32 (45.1%)	57 (37.5%)	0.28
LITA use (n, %)	66 (93.0%)	145 (95.4%)	0.52
Number of distal bypass (mean±SD)	2.51±0.93	2.67±0.89	0.18
Complete revascularization (n, %)	56 (78.9%)	128 (84.2%)	0.37
Aortic cross clamp time, minutes (mean±SD)	60.4±13.7	65.2±18.1	0.22
Total CPB time, minutes (mean±SD)	94.5±23.6	98.9±27.4	0.30
Total operation time, minutes (mean±SD)	209.5±44.3	217.6±50.8	0.51

CABG=coronary artery bypass grafting; CPB=cardiopulmonary bypass; LITA=left internal thoracic artery; SD=standard deviation

**Table 3:** Postoperative outcomes of the groups.

	Group 1 (Female)	Group 2 (Male)	*P* value
**Transfused blood products**			
Erythrocyte suspension, units (mean±SD)	1.70±0.55	1.42±0.83	0.21
Fresh frozen plasma, units (mean±SD)	2.06±0.77	1.89±0.95	0.12
Thrombocyte suspension, units (mean±SD)	0.21±0.03	0.23±0.04	0.67
Intubation time, hours (mean±SD)	9.3±4.4	8.7±5.9	0.40
ICU stay, hours (mean±SD)	44.0±59.2	36.7±33.7	0.24
Hospital stay, days (mean±SD)	12.1±13.2	10.4±8.6	0.25
**Complications**			
Low cardiac output syndrome (n,%)	3 (4.2%)	5 (3.3%)	0.86
Postoperative MI (n,%)	2 (2.8%)	3 (2.0%)	1.00
CVE (n,%)	3 (4.2%)	4 (2.6%)	0.68
Respiratory failure (n,%)	5 (7.0%)	6 (3.9%)	0.33
Pneumonia (n,%)	3 (4.2%)	4 (2.6%)	0.68
Reexploration for bleeding (n,%)	1 (1.4%)	2 (1.3%)	1.00
Mediastinitis (n,%)	4 (5.6%)	2 (1.3%)	0.08
Atrial fibrillation (n,%)	16 (22.5%)	41 (27.0%)	0.62
Acute renal dysfunction (n,%)	2 (2.8%)	4 (2.6%)	1.00
GIS complications (n,%)	1 (1.4%)	1 (0.7%)	0.53
In-hospital mortality (n,%)	2 (2.8%)	4 (2.6%)	1.00

CVE=cerebrovascular event; GIS=gastrointestinal system; ICU=intensive care unit; MI=myocardial infarction; SD=standard deviation

## DISCUSSION

Coronary artery disease was considered as a male disease and believed to affect almost exclusively men for many years. However, nowadays it is a very substantial health problem and the leading cause of mortality of female population in the United States, accounting for approximately 1 of every 3 female deaths^[16]^. CABG is used as an effective treatment modality for coronary artery disease in female patients, and females now account for up to 30% of patients undergoing myocardial revascularization procedures^[17]^. Incidence of morbidity and mortality during early postoperative period after CABG is shown to be higher in female patients than in male^[1-6]^. The most universal and contemporary operative risk score models include the gender as a significant parameter for predicting mortality after CABG^[18,19]^. However, studies evaluating independent impact of gender on early outcomes following CABG have presented variable results^[1-15]^. The impact of gender difference on early outcomes after CABG is controversial. In addition to this controversy, especially in elderly CABG patients, available data about the impact of gender on postoperative outcomes are not sufficient. Therefore, this study was designed to determine whether or not the gender difference affects the early postoperative outcomes in elderly CABG patients.

Our study showed no significant differences according to in-hospital mortality and major postoperative complications between females and males. Similary, Uncu et al.^[20]^ study on 174 participants aged above 75 years, concluded that CABG operations could be applied with similar mortality rates in women comparing to men. Arif et al.^[21]^ recent study on 4972 consecutive patients over 60 years revealed that women had a higher early postoperative mortality rate after CABG in only septuagenarians, but not in sexagenarians and octogenarians. Thus, the impact of gender difference on outcome may vary among different age groups. In a retrospective observational study on a total of 598 septuagenarian and octogenarian participants, no significant differences between genders were reported in terms of postoperative mortality and major complications such as central neurologic events, respiratory insufficiency, and renal failure requiring hemodialysis, thus female gender was not found to be associated with increased risks of morbidity and mortality after cardiac surgery in septuagenarians and octogenarians^[22]^. In another recent study performed by Berndt et al.^[23]^, the impact of gender difference on postoperative outcomes in octogenarians after CABG similarly reported that female gender was not associated with increased risks of morbidity and mortality. When the results of our study were compared to the results of these studies mentioned above, similar results according to the current literature could be found.

Although no statistically significant differences were found according to the postoperative complications, the rate of development of mediastinitis in female patients was 4.3 times higher than males. We believe that this difference was not statistically significant (5.6% *vs.* 1.3%, *P*=0.08), probably due to the small sample size of our study population. In a study performed by Sá et al.^[24]^, the risk factors for mediastinitis after CABG were studied, and obesity, diabetes, smoking, use of pedicled internal thoracic artery and on-pump CABG were determined as independent risk factors of mediastinitis after CABG.

Almost all studies in the literature reported that female patients undergoing CABG were older and higher risk patients with higher EuroSCORE had more preoperative comorbid conditions such as HT, DM and HL compared to male^[25-28]^. We also found that female patients had statistically significantly higher EuroSCORE levels, and the incidence of HT and HL were higher than in males. The incidence of DM was also higher in the female group, but the difference of incidence of diabetes mellitus between the groups was not statistically significant (38% *vs.* 26.3%; *P*=0.06). As a result, according to the literature, our study population had also similar features in terms of preoperative patients' demographics.

Some surgeons can behave timidly to apply a CABG operation for female patients, and a delayed decision for operation may be a reason of disease progression, increased surgical risk, decreased long-term survival, and higher incidence of comorbid disorders^[29]^. Therefore, a consensus establishment regarding when to apply a CABG operation for female patients is crucial. Additionally, detailed anamnesis, rigorous physical examination, laboratory tests and radiological imaging methods can assist to specify the physiological reserves and optimal conditions of the patients for CABG^[20]^.

LITA is the most commonly used arterial graft in CABG accounting for its satisfactory patency rate, convenience for coronary artery anastomosis when it does not need proximal anastomosis. The importance of LITA graft use was emphasized in numerous reports, and the LITA graft use had proven to decrease the mortality rates and increase long-term survival in both male and female CABG patients^[30-32]^. Therefore, LITA as coronary bypass graft in our practice is commonly preferred. In this series, we used LITA grafts in 93% of females and 95.4% of males (94.6% of overall), but the difference between the groups was not statistically significant (*P*=0.52). Despite no statistically significant difference, similar to our study, the majority of studies in the literature has documented that the LITA graft use is less common in female patients.

## CONCLUSION

The results of this present study will ensure some clarity to this controversial subject as no statistically significant differences were found in early postoperative outcomes in elderly female and male patients. However, there were several limitations in the interpretation of the results of our study. The major limitations of this study were the retrospective nature of data collection, evaluated data were limited, lack of the mid and long-term outcomes of patients, and relatively small number of patients in the study groups. A larger sample could have increased the statistical power of our research. This study demonstrated that the female gender was not associated with worse early postoperative outcomes in elderly CABG patients.

**Table:** 

Authors' roles & responsibilities	
AY	Substantial contributions to the conception or design of the work; or the acquisition, analysis, or interpretation of data for the work; drafting the work or revising it critically for important intellectual content; final approval of the version to be published
IIK	Substantial contributions to the conception or design of the work; or the acquisition, analysis, or interpretation of data for the work; final approval of the version to be published
AY	Substantial contributions to the conception or design of the work; or the acquisition, analysis, or interpretation of data for the work; drafting the work or revising it critically for important intellectual content; final approval of the version to be published
YV	Substantial contributions to the conception or design of the work; or the acquisition, analysis, or interpretation of data for the work; drafting the work or revising it critically for important intellectual content; final approval of the version to be published
MÇÇ	Substantial contributions to the conception or design of the work; or the acquisition, analysis, or interpretation of data for the work; drafting the work or revising it critically for important intellectual content; final approval of the version to be published
OG	Substantial contributions to the conception or design of the work; or the acquisition, analysis, or interpretation of data for the work; drafting the work or revising it critically for important intellectual content; final approval of the version to be published
GK	Agreement to be accountable for all aspects of the work in ensuring that questions related to the accuracy or integrity of any part of the work are appropriately investigated and resolved; final approval of the version to be published
SA	Agreement to be accountable for all aspects of the work in ensuring that questions related to the accuracy or integrity of any part of the work are appropriately investigated and resolved; final approval of the version to be published
MB	Agreement to be accountable for all aspects of the work in ensuring that questions related to the accuracy or integrity of any part of the work are appropriately investigated and resolved; final approval of the version to be published
MT	Drafting the work or revising it critically for important intellectual content;
IŞ	Substantial contributions to the conception or design of the work; or the acquisition, analysis, or interpretation of data for the work; final approval of the version to be published
